# The evolution of data treatment tools in single-particle and single-cell ICP-MS analytics

**DOI:** 10.1007/s00216-024-05513-4

**Published:** 2024-09-04

**Authors:** Michail Ioannis Chronakis, Björn Meermann, Marcus von der Au

**Affiliations:** Division 1.1 - Inorganic Trace Analysis, Federal Institute for Materials Research and Testing (BAM), Richard-Willstätter-Straße 11, 12489 Berlin, Germany

**Keywords:** Mass spectrometry/ICP-MS, Cell systems/single-cell analysis, Nanoparticles/nanotechnology, Data treatment

## Abstract

Single-particle inductively coupled plasma-mass spectrometry (sp-ICP-MS) is one of the most powerful tools in the thriving field of nanomaterial analysis. Along the same lines, single-cell ICP-MS (sc-ICP-MS) has become an invaluable tool in the study of the variances of cell populations down to a per-cell basis. Their importance and application fields have been listed numerous times, across various reports and reviews. However, not enough attention has been paid to the immense and ongoing development of the tools that are currently available to the analytical community for the acquisition, and more importantly, the treatment of single-particle and single-cell-related data. Due to the ever-increasing demands of modern research, the efficient and dependable treatment of the data has become more important than ever. In addition, the field of single-particle and single-cell analysis suffers due to a large number of approaches for the generated data—with varying levels of specificity and applicability. As a result, finding the appropriate tool or approach, or even comparing results, can be challenging. This article will attempt to bridge these gaps, by covering the evolution and current state of the tools at the disposal of sp-ICP-MS users.

**Graphical Abstract:**

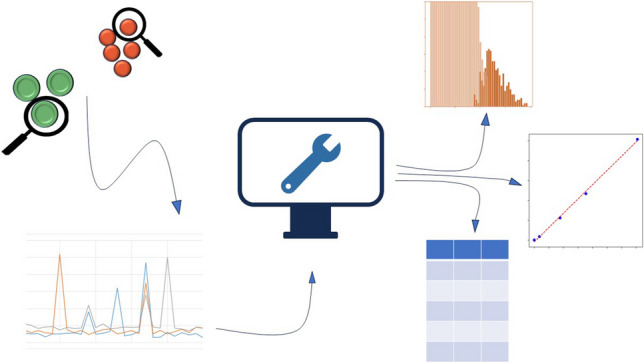

## Introduction

Inductively coupled plasma-mass spectrometry (ICP-MS) can be traced back to 1980 [1] and it continues to be an invaluable tool for a plethora of analytical sub-fields and applications [2]. One of these sub-fields is the investigation of small entities, as a whole, in the nano- and micrometre scale: the so-called single-particle ICP-MS (sp-ICP-MS) and single-cell ICP-MS (sc-ICP-MS) [3]. sp-ICP-MS is an efficient tool in the determination of the size, number concentration, and elemental content determination of nanoparticles [4]. sc-ICP-MS on the other hand is usually focused on the study of the heterogeneity within cell populations. Through the study of intrinsic elements and/or metal tags within the cells, the study of genes, proteins, and their expression is enabled, down to a single-cell level [5]. Despite their different objectives, the principle of operation is quite similar between sp- and sc-ICP-MS: After suitable dilution, the suspension is introduced into the ICP-MS, at which point the entity is atomized into the so-called *ion cloud*. The ion cloud passes through the instrumentation, reaching the detector in its entirety, in a short time window. The detector is set on a comparable collection window per produced data point, generating, in the presence of a single entity within the collection window, what is called an *event*. Several reviews have since directly addressed the principle of operation, its benefits and shortcomings, the wide range of application fields as well as its critical aspects, and its importance to the future of micro- and nanoscale analysis [3, 6–9].

The rising interest of the community led to extensive fine-tuning and tailoring of the technique to the needs of the samples. The temporal resolution was one of the first aspects that was further explored. As a result, lower dwell times (monitoring time per data point, per *m*/*z*) were incorporated, aiming to increase the signal-to-noise ratio of the detected events (from ms to µs, even ns range) [10, 11]. As the dwell time became shorter than the duration of an ion cloud (300–1000 µs) [12, 13], events stretched from one data point per event into several. The term “transient signals” was allocated for the collection of data points on lower dwell times. From a treatment point of view, data points belonging to transient events needed not only be identified (e.g., separated from the background), but also be correctly grouped to their respective events. In that manner, the particle number can be more accurately estimated. Moreover, it was observed that the determination of the critical values used in the identification of the signals, meaning, the decision threshold, was also occasionally inaccurate during the use of lower dwell times. This was at the time attributed to the different baseline distributions exhibited for low-intensity signals, which is vital for the calculation of the critical values. That fact added one more step to the decision of the critical values, e.g., identifying the background distribution [10, 11, 14]. In addition, the quantification process of the individual events changed. In the case of a single data point per event, the height of the intensity signal is correlated to the size of the entity detected. In the case of transient events, the integral (or sum) of the data points comprising the event needs to be considered instead [10, 15–17]. Furthermore, the study of multiple elements in single entities highlighted the need for the acquiring of multi-elemental profiles, as this would enable the fingerprinting of the detected entities. For those purposes, the time-of-flight (TOF) mass analyser was incorporated, using a multichannel plate (MCP) detector assembly, for the quasi-simultaneous detection of multiple elements in individual events (sp/sc-ICP-TOFMS). However, due to the nature of the MCP, the baseline distribution does not follow the normal, lognormal, or Poisson distributions that were previously observed for other detector types, like electron multipliers used for quadruple-based instruments. Therefore, the process for the determination of the critical values for the detection of single entities used up to that point would prove insufficient. Later on, the MCP-generated background distribution was successfully modelled as a compound Poisson distribution through the use of a Monte Carlo simulation approach, and a more appropriate way to determine the critical values was described [18–20]. In addition, that process needs to happen individually for every *m*/*z* of interest, increasing either the time of the data-treating process, or the processing power needed, or both. Similarly, the information sought via a multi-elemental analysis is often derived by the correlation of the results of different isotopes, via processes like Principal Component Analysis (PCA), which adds to the workload of the data treatment approach. Finally, the size of the datasets is increasing with the addition of more isotopes of interest, further indicating the need for the upgrade of the equipment involved or the process of the data treatment [19]. Following, therefore, along the evolution of the sp-ICP-MS over the years, one can easily spot the difficulties stemming from the increasing relative size of the acquired datasets, as well as the increase in the complexity of the necessary data treatment and evaluation. Even though the results and applications have been highlighted in the previously published reviews, up until recently, little attention has been allocated to pointing out the extensive efforts of the analytical community, in terms of keeping up with the increasing demands of the acquired data. Within this article, the progression and advancements of data treatment and evaluation tools in the field of sp/sc-ICP-MS, including sp/sc-ICP-TOFMS are highlighted.

## Advancements in data treatment tools

Overall, the features of the data treatment tools include either sample information, like particle number density, mass or size, or signal deconvolution options, or both. After the introduction of the multi-element single-particle analysis, the data treatment tools were likewise upgraded. In order to avoid any kind of conflicting interest, the vendor software options have been neglected within this article. However, it is noteworthy that most vendors have their proprietary options for the treatment of single-particle and single-cell-related data. They all prioritize user-friendly interfaces, smoothing their learning curve. On the other hand, they usually lack in terms of transparency, as it is unlikely for a company to divulge the inner workings of their algorithms, and flexibility, as the available options are usually specific.

### Text-based interface approaches and non-specialized options

Microsoft Excel has been utilized in the early stages, owing to its universal availability and popularity [21]. In addition, it is purposefully user-friendly, enabling easy copy-and-paste solutions which are familiar to most users. Furthermore, much has been proven achievable through the incorporation of its numerous functions, and/or VBA (Visual Basic for Applications), a coding-like implementation into the Microsoft environment. “SPC” is one of the first tools that was widely available to the community and is based on Microsoft Excel. The nowadays called “SPC,” for “single-particle calculation” spreadsheet (last updated in 2015, available for download: https://www.wur.nl/en/show/single-particle-calculation-tool.htm), was developed by RIKILT of the Wageningen University and Research Centre. While it needed a manual input for the threshold of the particle detection, meaning it did not include signal treatment options, it offered a step towards automatization of the data treatment process by calculating relevant information about the input data. More recently, Laborda et al. developed a different spreadsheet that includes all the calculations necessary for the estimation and prediction of event identification threshold criteria (available for download: directly from the journal’s page on the article) [14].

The aforementioned increased complexity of the generated data led to other available options being explored, that featured higher processing power or ability to handle larger datasets, which has always been a known limitation with Excel. For instance, Igor Pro 6.37 has been incorporated in the treatment of low-dwell time acquisitions (75 µs), complementary to Microsoft Excel, for peak identification, integration, and calculation of the necessary sample information [22]. In the case of more complex treatment, like the multivariate analysis of multi-elemental datasets, OriginPro has also been reportedly incorporated in the field of single-cell analysis, as a complementary tool after the signal deconvolution has been done by vendor software [23].

Programming languages have also played a major role in modern signal analytics, owing to their flexibility and wide variety of capabilities. The implementation of that versatility enables even the potential automatization of the data-treating process, saving in terms of time and energy. The general preference turns to high-level languages (farther from the “machine” language and closer to human experience), like Python or C. As an example, for the needs of the fast data generated by their home-built data acquisition unit, Engelhard et al. have been utilizing “C” [11, 24, 25]. A dedicated command line tool has been developed for the processing of the raw data, designed to extract any information related to particles. Taking advantage of the aforementioned flexibility, programming is becoming key in the field of multi-elemental analytics. Mehrabi et al. performed their sp-ICP-TOFMS analysis solely on MATLAB, on a quite complex experimental paradigm, involving a microdroplet generator on-line coupled to a conventional ICP-TOFMS introduction system [26]. The program was designed to identify microdroplet signals by their (known) multi-elemental fingerprint, while also running the signal deconvolution procedure for the nanoparticles, using the compound Poisson modelling mentioned earlier. Python code has also been reported to be used exclusively as a signal deconvolution and sample information tool in the multi-elemental analysis of single cells, in an AF4/ICP-TOFMS approach [27]. The code was designed to extract events for every chosen element, identify events that contained more than one element, and give information about number density, relative ratios of elements, etc.

### Graphic user interface approaches

The first graphic user interface (GUI) option, NanoCount, was developed by Cornelis et al., during a process that stretches over several years (available for download: https://blogg.slu.se/nanocount/). The first version of NanoCount came to be back in 2014, when it was not a GUI yet, but a piece of code written in MATLAB, yet one of the first automated tools for signal deconvolution [28]. The first interactive version of the software came after the publication of a drift-correction approach by Cornelis et al. [29]. Since 2017, the third and improved version has been available to the public, featuring options for signal deconvolution in both single- and multiple-data points per nanoparticle datasets, as well as background drift correction and transport efficiency determination. The output options include treated data, as well as histograms and relevant values like particle concentration, etc.

SPCal is a more recently developed option for the handling of single-particle data. It is a Python-based interactive platform, freely available to users as a self-contained executable file (download: https://github.com/djdt/spcal). The open-source logic promotes transparency in terms of the incorporated algorithms, to go along with the user-friendliness of a graphic user interface. Since its beginning, it accepted a variety of relevant file types, and it featured signal deconvolution options in accordance with the latest developments in the field. Regarding the outputs, it returns all relevant sample information, as well as optional exported images of the relevant histograms. There are also options for the optimal fitting of the data (normal, lognormal, etc.), as well as the exporting of the treated data. With its newest version available, it is rapidly evolving, and can nowadays accept sp/sc-ICP-MS as well as sp/sc-ICP-TOFMS data, while its processing can give out relevant histograms and fitted distributions, scatter and PCA plots, and more [30].

TOF-SPI (“TOF Single Particle Investigator”) is specialized to sp/sc-ICP-TOFMS generated data. It is developed in LabVIEW, and it is freely available as a self-contained executable file (available for download: https://github.com/TOFMS-GG-Group). It is capable of batch analysis and designed for optional calibration with an online microdroplet generator. Developed by the same group that published the newest approach for the determination of the baseline as a compound Poisson distribution in TOF instruments, it uses that same algorithm to calculate threshold values for the identification of particle events. It features several useful functions, like identifying and correcting split particle cases, background-correcting the data, etc. It generates output on several related variables, including absolute sensitivities, background information, as well as information about the mass of the user-chosen analytes. It is also able to handle the size conversion, in the case of particles, following the established procedures [31]. The approaches mentioned in this chapter are summarized in Table 1.

**Table 1:** Grouped data treatment approaches, with their subjective advantages and disadvantages

Approach	Advantages	Disadvantages	Application examples
Spreadsheet-based software (Excel, Origin, etc.)	✓ Smooth learning curve ✓ Widespread use ✓ Good for quick results/screening	× Limited capabilities, especially in multi-elemental analysis × Difficult to implement iterative processes × Not many advanced treatment options	• Monitoring the fate of ultratrace levels of dissolved Ag^+^ and Ag NPs in seawater systems [22] • Monitoring the interactions of Cd, Ce, and U with individual spores [32]
Independently developed software	✓ Open-source ✓ Transparent processes ✓ Advanced treatment options	× Less user-friendly design × Potentially steep learning curve × Often discontinued	• TiO_2_ NPS detection in Ca-rich matrices (NanoCount) [33] • Analysis of Pb and Ti NPs in the aqueous environment of Melbourne (SPCal) [34]
Programming options	✓ Complete control over the treatment process ✓ Advanced treatment available ✓ No limitations	× No user interface × Steep learning curve × Developed approaches usually are fit-for-purpose	• Detection of engineered Cu NPs (MATLAB) [35] • Multi-elemental analysis and simultaneous quantification of particles from difficult matrices (MATLAB) [26]

## Machine learning approaches

The field of single-particle analytics has been rapidly evolving, at an increasing rate, and the development of data treatment tools and options is likewise evolving as best as humanly possible. It is not, however, unrealistic to believe that there might soon come a day when information might go unnoticed, due to inadequacy in terms of extracting it from the acquired data, simply due to human inability to properly identify the emerging pattern. For that purpose, the analytical community has already begun taking advantage of the possibilities being made available through the use of machine learning algorithms [36]. Machine learning (ML) is an umbrella term, part of the evolving field of artificial intelligence (AI). Its purpose has always been to solve problems in a way that would be more time and cost efficient than a human alternative, basically by developing its own algorithmic processes. This happens by first “training” the program in the way that the problem needs to be solved, and then allowing it to apply its acquired “knowledge” to specific examples. After appropriate training, the developed algorithm should also be able to eliminate the possibility of human error related to the performing of necessary, repeated tasks. Based on this paradigm, there are three broad categories into which ML approaches can be divided: (i) In the so-called “supervised learning” approach, the program is given examples of input data, with their respective desired outputs, and its goal is to make the logical connections and develop its way to extract similar outputs of similar inputs. (ii) “Unsupervised learning” differs in the way that there is no guidance from the user, as the name suggests. Rather, the data are given to the program, which is designed to identify the patterns on its own, which sometimes is itself the task at hand. (iii) As a third approach, there is “reinforcement learning,” which includes the program operating on a task-feedback loop, to navigate a situation, trying to optimize or maximize a specific aspect of that work (f.i. driving a car). While it is true that no approach is fit for every situation, there is one thing that AI will always perform better at than the vast majority of humans: pattern recognition [37].

Supervised approaches have already been used in a variety of ways in the single-particle data treatment process. For instance, a supervised gradient decision tree boosting classification (GBC) has been used in the differentiation process between engineered and natural nanomaterials [38]. The training process of the model starts with giving it sp-ICP-TOFMS data of reference material, at which point it “learns” the process of identifying them, by choosing the parameters that contribute to the differentiation the most. The configuration of the model is considered optimum when it produces the highest median performance, using the lowest number of parameters. The then-trained model is validated through the pristine sample analysis and then applied to the real samples. Another similar approach has been incorporated in the distinction between engineered TiO_2_ from natural Ti nanomaterials in soil, using binomial logistic regression (BL) [39]. It was similarly trained by giving reference samples of mixed engineered and natural material. Supervised learning has also been incorporated in the analysis of airborne particles [40], while another TOF-related approach utilized a laser ablation introduction system to analyse road dust samples. In that case, a supervised classification approach was used to create a potentially fully automatized data processing pipeline [41].

On the other hand, unsupervised machine learning, as was already mentioned, is a very suitable option for identifying previously unknown patterns. For that reason, it has been incorporated into fingerprinting natural nanomaterials through the analysis of their multi-elemental profile (sp-ICP-TOFMS) [42]. Advancing the currently used methods, unsupervised clustering analysis has been incorporated in the classification of particles in wastewater treatment samples, focusing on the immense potential that the full automatization of the process exhibits [43]. In both of those cases, data have been given to the developed model, which was itself tasked with identifying the emerging patterns, among other things. Furthermore, a multi-staged, semi-supervised strategy has recently been developed for the classification of Ce-containing particles as natural, incidental, or engineered [44]. The ultimate goal of this study was to mitigate the systematic discrimination that TOFMS instruments exhibit when it comes to the moiety’s elemental composition, related to their size. Not only was the implemented strategy able to make the distinction with confidence, but it also provided the tools for the better identification of individual events that were previously not safely extracted from their background.

Despite their numerous potential advantages, machine learning approaches are highly dependent upon their training process. ICP-MS, and especially sp/sc-ICP-MS, is a complex technique, with numerous variables involved. Understanding the nature of the baseline signal can be critical, and it is a vital part of the threshold criterion choosing process, which is the cornerstone of the data-treating process [10, 14, 19, 45]. In addition, factors like variable sensitivities, elemental composition of the entity, and even similarities between training “samples” or datasets with real ones, etc. should all be considered during the model training process. Furthermore, the computer running the training lacks any kind of inherent critical thinking. That bears the risk that the in-process feedback can be misinterpreted, as the computer will only check for the parameters it is instructed to, before moving to the next iteration. Therefore, caution must be exercised when developing a machine learning approach, and a strict process for the validation of the training method should be incorporated before the results are considered dependable.

## Outlook

The options of data-treating tools, when it comes to single-particle and single-cell ICP-MS analysis, have been steadily evolving over the past few years. The ever-increasing incorporation of the technique in a variety of fields has led to a proportionate increase in the demands of the community regarding their capabilities. The limitations of traditional means of data evaluation, like spreadsheets and other commercial multi-purpose software, have led to the development of more specialized solutions. This manifested in the development of both novel tools, specially designed for the respective task, and the incorporation of programming into the data treatment and evaluation process.

Nowadays, however, it is becoming clear, that the evolution and demands of the field might soon outpace the capabilities of human-driven analysis, regarding the obtaining of the maximum amount of information from the analytical process. As a result, the first artificial intelligence options have long since made their appearance and have occasionally proven superior to their manual counterparts. On the other hand, the successful incorporation of machine learning approaches, in the field of data treatment in the field of single-particle and single-cell ICP-MS, raises concerns about the quality management of the AI-generated results, and they need to be addressed before this tool can be further implemented in the analytical process.

The ultimate goal seems to be the automatization of an increasing portion of the whole analytical process, from the sample obtaining to treating the data. Thus, the capacity for the currently irreplaceable human scientific sight, intuition, and improvisation can be increased, allowing for the focusing on generating the questions, instead of attending to laborious and repetitive processes. As the analytical field evolves, so will the capabilities, and therefore, the potential benefits to every other field and discipline, both current and potentially still unrealized. The field of multi-elemental single-entity analytics, in particular, is at the moment largely underappreciated. The single-cell metallomics field has been gaining in momentum, highlighting its importance, and indicating its potential future applications. For instance, model cell organisms have been incorporated in the ecotoxicological assessment of natural systems. Furthermore, the root cause of many diseases can be linked to their elemental content, and the relative levels of different target analytes. A streamlined nanomaterial fingerprinting process, from sample introduction to data evaluation, could be of great benefit in numerous fields like forensics or archaeometry; for example, the multi-elemental profiling of archaeological samples can lead to the identification of previously unobserved patterns and correlations. The successful and dependable fingerprinting of gunpowder residue can be an invaluable tool in the field of forensics. In all of the above-mentioned cases, the treatment of the data will prove to be equally as important as the sound technical knowledge and application of the instrumentation, so that the integrity of the results can be safeguarded. Therefore, the evolution of the available data treatment options should be closely monitored, in order to create dependable tools that address the needs of the analytical procedures.

## References

[1] Houk RS, Fassel VA, Flesch GD, Svec HJ, Gray AL, Taylor CE. Inductively coupled argon plasma as an ion source for mass spectrometric determination of trace elements. Anal Chem. 1980;52(14):2283–9.

[2] Van Acker T, Theiner S, Bolea-Fernandez E, Vanhaecke F, Koellensperger G. Inductively coupled plasma mass spectrometry. Nat Rev Methods Primers. 2023;3(1):52.

[3] Resano M, Aramendía M, García-Ruiz E, Bazo A, Bolea-Fernandez E, Vanhaecke F. Living in a transient world: ICP-MS reinvented via time-resolved analysis for monitoring single events. Chem Sci. 2022;13(16):4436–73. 35656130 10.1039/d1sc05452j PMC9020182

[4] Laborda F, Bolea E, Jiménez-Lamana J. Single particle inductively coupled plasma mass spectrometry: a powerful tool for nanoanalysis. Anal Chem. 2014;86(5):2270–8. 24308527 10.1021/ac402980q

[5] Theiner S, Loehr K, Koellensperger G, Mueller L, Jakubowski N. Single-cell analysis by use of ICP-MS. J Anal At Spectrom. 2020;35(9):1784–813.

[6] Montaño MD, Olesik JW, Barber AG, Challis K, Ranville JF. Single particle ICP-MS: advances toward routine analysis of nanomaterials. Anal Bioanal Chem. 2016;408(19):5053–74. 27334719 10.1007/s00216-016-9676-8

[7] Meermann B, Nischwitz V. ICP-MS for the analysis at the nanoscale – a tutorial review. J Anal At Spectrom. 2018;33(9):1432–68.

[8] Mozhayeva D, Engelhard C. A critical review of single particle inductively coupled plasma mass spectrometry – a step towards an ideal method for nanomaterial characterization. J Anal At Spectrom. 2020;35(9):1740–83.

[9] Bolea E, Jimenez MS, Perez-Arantegui J, Vidal JC, Bakir M, Ben-Jeddou K, et al. Analytical applications of single particle inductively coupled plasma mass spectrometry: a comprehensive and critical review. Anal Methods. 2021;13(25):2742–95. 34159952 10.1039/d1ay00761k

[10] Abad-Álvaro I, Peña-Vázquez E, Bolea E, Bermejo-Barrera P, Castillo JR, Laborda F. Evaluation of number concentration quantification by single-particle inductively coupled plasma mass spectrometry: microsecond vs millisecond dwell times. Anal Bioanal Chem. 2016;408(19):5089–97. 27086011 10.1007/s00216-016-9515-y

[11] Strenge I, Engelhard C. Capabilities of fast data acquisition with microsecond time resolution in inductively coupled plasma mass spectrometry and identification of signal artifacts from millisecond dwell times during detection of single gold nanoparticles. J Anal At Spectrom. 2016;31(1):135–44.

[12] Fuchs J, Aghaei M, Schachel TD, Sperling M, Bogaerts A, Karst U. Impact of the particle diameter on ion cloud formation from gold nanoparticles in ICPMS. Anal Chem. 2018;90(17):10271–8. 30056707 10.1021/acs.analchem.8b02007

[13] Montaño MD, Badiei HR, Bazargan S, Ranville JF. Improvements in the detection and characterization of engineered nanoparticles using spICP-MS with microsecond dwell times. Environ Sci Nano. 2014;1(4):338–46.

[14] Laborda F, Gimenez-Ingalaturre AC, Bolea E, Castillo JR. About detectability and limits of detection in single particle inductively coupled plasma mass spectrometry. Spectrochim Acta Part B. 2020;169:105883.

[15] Kálomista I, Kéri A, Ungor D, Csapó E, Dékány I, Prohaska T, et al. Dimensional characterization of gold nanorods by combining millisecond and microsecond temporal resolution single particle ICP-MS measurements. J Anal At Spectrom. 2017;32(12):2455–62.

[16] Liu J, Wei X, Wu C, Zheng L, Wang M, Chen M, et al. Data analysis for the characterization of nanoparticles with single particle inductively coupled plasma mass spectrometry: from microsecond to millisecond dwell times. Anal Chim Acta. 2023;1254:341114. 37005024 10.1016/j.aca.2023.341114

[17] Laborda F, Abad-Álvaro I, Jiménez MS, Bolea E. Catching particles by atomic spectrometry: benefits and limitations of single particle - inductively coupled plasma mass spectrometry. Spectrochim Acta Part B. 2023;199:106570.

[18] Gundlach-Graham A. Chapter Three - Multiplexed and multi-metal single-particle characterization with ICP-TOFMS. In: Milačič R, Ščančar J, Goenaga-Infante H, Vidmar J, editors. Comprehensive analytical chemistry. 93. Amsterdam: Elsevier; 2021. p. 69–101.

[19] Gundlach-Graham A, Hendriks L, Mehrabi K, Günther D. Monte Carlo simulation of low-count signals in time-of-flight mass spectrometry and its application to single-particle detection. Anal Chem. 2018;90(20):11847–55. 30240561 10.1021/acs.analchem.8b01551

[20] Hendriks L, Gundlach-Graham A, Günther D. Performance of sp-ICP-TOFMS with signal distributions fitted to a compound Poisson model. J Anal At Spectrom. 2019;34(9):1900–9.

[21] Mitrano DM, Lesher EK, Bednar A, Monserud J, Higgins CP, Ranville JF. Detecting nanoparticulate silver using single-particle inductively coupled plasma-mass spectrometry. Environ Toxicol Chem. 2012;31(1):115–21. 22012920 10.1002/etc.719

[22] Chronakis MI, Mavrakis E, García RÁ-F, Montes-Bayón M, Bettmer J, Pitta P, Tsapakis M, et al. Investigating the behavior of ultratrace levels of nanoparticulate and ionic silver in a seawater mesocosm using single particle inductively coupled plasma – mass spectrometry. Chemosphere. 2023;336:139109. 37270041 10.1016/j.chemosphere.2023.139109

[23] von der Au M, Borovinskaya O, Flamigni L, Kuhlmeier K, Büchel C, Meermann B. Single cell-inductively coupled plasma-time of flight-mass spectrometry approach for ecotoxicological testing. Algal Res. 2020;49:101964.

[24] Mozhayeva D, Strenge I, Engelhard C. Implementation of online preconcentration and microsecond time resolution to capillary electrophoresis single particle inductively coupled plasma mass spectrometry (CE-SP-ICP-MS) and its application in silver nanoparticle analysis. Anal Chem. 2017;89(13):7152–9. 28602085 10.1021/acs.analchem.7b01185

[25] Strenge I, Engelhard C. Single particle inductively coupled plasma mass spectrometry: investigating nonlinear response observed in pulse counting mode and extending the linear dynamic range by compensating for dead time related count losses on a microsecond timescale. J Anal At Spectrom. 2020;35(1):84–99.

[26] Mehrabi K, Günther D, Gundlach-Graham A. Single-particle ICP-TOFMS with online microdroplet calibration for the simultaneous quantification of diverse nanoparticles in complex matrices. Environ Sci Nano. 2019;6(11):3349–58.

[27] Chronakis MI, von der Au M, Meermann B. Single cell-asymmetrical flow field-flow fractionation/ICP-time of flight-mass spectrometry (sc-AF4/ICP-ToF-MS): an efficient alternative for the cleaning and multielemental analysis of individual cells. J Anal At Spectrom. 2022;37(12):2691–700.

[28] Cornelis G, Hassellöv M. A signal deconvolution method to discriminate smaller nanoparticles in single particle ICP-MS. J Anal At Spectrom. 2014;29(1):134–44.

[29] Cornelis G, Rauch S. Drift correction of the dissolved signal in single particle ICPMS. Anal Bioanal Chem. 2016;408(19):5075–87. 27095581 10.1007/s00216-016-9509-9

[30] Lockwood TE, Gonzalez de Vega R, Clases D. An interactive Python-based data processing platform for single particle and single cell ICP-MS. J Analyt Atom Spectrom. 2021;36(11):2536–44.

[31] Gundlach-Graham A, Harycki S, Szakas SE, Taylor TL, Karkee H, Buckman RL, et al. Introducing “Time-of-Flight Single Particle Investigator” (TOF-SPI): a tool for quantitative spICP-TOFMS data analysis. J Anal Atom Spectrom. 2024;39:704–11.

[32] Hellmann S, García-Cancela P, Alonso-Fernández S, Corte-Rodríguez M, Bettmer J, Manteca A, et al. Single cell ICP-MS to evaluate the interaction behaviour for Cd, Ce and U with Streptomyces coelicolor spores. Chemosphere. 2024;347:140633. 37951404 10.1016/j.chemosphere.2023.140633

[33] Tharaud M, Gondikas AP, Benedetti MF, von der Kammer F, Hofmann T, Cornelis G. TiO2 nanomaterial detection in calcium rich matrices by spICPMS. A matter of resolution and treatment. J Anal Atom Spectrom. 2017;32(7):1400–11.

[34] Gonzalez de Vega R, Lockwood TE, Xu X, Gonzalez de Vega C, Scholz J, Horstmann M, et al. Analysis of Ti- and Pb-based particles in the aqueous environment of Melbourne (Australia) via single particle ICP-MS. Anal Bioanal Chem. 2022;414(18):5671–81. 35482065 10.1007/s00216-022-04052-0 PMC9242955

[35] Navratilova J, Praetorius A, Gondikas A, Fabienke W, Von der Kammer F, Hofmann T. Detection of engineered copper nanoparticles in soil using single particle ICP-MS. Int J Environ Res Public Health. 2015;12(12):15756–68. 26690460 10.3390/ijerph121215020 PMC4690956

[36] Wu ZQ, Ma YP, Liu H, Huang CZ, Zhou J. High confidence single particle analysis with machine learning. Anal Chem. 2023;95(41):15375–83. 37796610 10.1021/acs.analchem.3c03297

[37] Bishop CM. Pattern recognition and machine learning. New York: Springer; 2006.

[38] Praetorius A, Gundlach-Graham A, Goldberg E, Fabienke W, Navratilova J, Gondikas A, et al. Single-particle multi-element fingerprinting (spMEF) using inductively-coupled plasma time-of-flight mass spectrometry (ICP-TOFMS) to identify engineered nanoparticles against the elevated natural background in soils. Environ Sci Nano. 2017;4(2):307–14.

[39] Bland GD, Battifarano M, Pradas del Real AE, Sarret G, Lowry GV. Distinguishing engineered TiO2 nanomaterials from natural Ti nanomaterials in soil using spICP-TOFMS and machine learning. Environ Sci Technol. 2022;56(5):2990–3001. 35133134 10.1021/acs.est.1c02950

[40] Bland GD, Battifarano M, Liu Q, Yang X, Lu D, Jiang G, et al. Single-particle metal fingerprint analysis and machine learning pipeline for source apportionment of metal-containing fine particles in air. Environ Sci Technol Lett. 2022;10:1023–9.

[41] Holbrook TR, Gallot-Duval D, Reemtsma T, Wagner S. Machine learning: our future spotlight into single-particle ICP-ToF-MS analysis. J Anal At Spectrom. 2021;36(12):2684–94.

[42] Baalousha M, Wang J, Erfani M, Goharian E. Elemental fingerprints in natural nanomaterials determined using SP-ICP-TOF-MS and clustering analysis. Sci Total Environ. 2021;792:148426. 34157530 10.1016/j.scitotenv.2021.148426

[43] Mehrabi K, Kaegi R, Günther D, Gundlach-Graham A. Emerging investigator series: automated single-nanoparticle quantification and classification: a holistic study of particles into and out of wastewater treatment plants in Switzerland. Environ Sci Nano. 2021;8(5):1211–25. 34046179 10.1039/d0en01066a PMC8136323

[44] Buckman RL, Gundlach-Graham A. Machine learning analysis to classify nanoparticles from noisy spICP-TOFMS data. J Anal At Spectrom. 2023;38(6):1244–52.

[45] Laborda F, Gimenez-Ingalaturre AC, Bolea E, Castillo JR. Single particle inductively coupled plasma mass spectrometry as screening tool for detection of particles. Spectrochim Acta Part B. 2019;159:105654.

